# Our New York Letter

**Published:** 1890-12

**Authors:** Wm. L. Russell

**Affiliations:** New York


### Correspondence.

### OUR NEW YORK LETTER.

New York, November 15, 1890.

### ACUTE PLEURISY.

An editorial article in the last number of the *Medical Record* discusses the interesting question of the treatment of acute pleurisy from cold with salicylic acid. The ground on which this plan of treatment is based is the similarity between such cases and those of acute rheumatism. It is argued that exposure to cold is often regarded as the exciting cause of both, and that both are accompanied by serous effusions, in the one case into the pleural cavity, and in the other into joint cavities, the lesser effusion in the latter instance being the result of the greater resistance of the tissues about the joints. The pleura may be thus regarded as a joint, and its acute inflammation may be due to the identical cause which produces acute rheumatism. These views being accepted, the usefulness of salicylic acid would become highly probable. The difficulty would be to decide the matter practically.

### PAGE ON PHYSICAL DIAGNOSIS.

One of the best books on the physical diagnosis of diseases of the chest which has lately appeared is that of Professor Page. In about a year and a half two editions have been issued, and the second is now nearly exhausted. Those who have heard Professor Page at the Polyclinic will recognize in his book the same directness and simplicity of expression, the same earnest enthusiasm, and the same fidelity to simple practical trnth which render him so popular as a lecturer and so helpful as a teacher.

The book is probably more in keeping with the modern scientific spirit than that of any other American writer on the subject. The reason for each physical sign is explained with logical clearness, and a lasting impression is thus made on the mind of the student. The work is by no means as exhaustive as those of Gutmann and other foreign authors, but it is sufficiently full for all practical purposes. The illustrations are profuse and well executed, being of real assistance to an understanding of the text. It is on the whole the best book on the subject which has yet been published in America.

### PURPURA HEMORRHAGICA.

At the last meeting of the Academy, an interesting paper on purpura hemorrhagica and its relation to other hemorrhagic diseases was read by Dr. G. R. Lockwood. The article opened with a reference to the recent discovery of a bacillus which it was claimed produced the disease when injected into rabbits. Then followed the record of a case which resulted in death after two and a half days' illness. The disease was ushered in suddenly by two chills, which were followed by uncontrollable nosebleed. Purpuric spots then appeared in the skin, and hemorrhage from the uterus and bowels soon followed. The spots increased and the hemorrhage continued. The patient became somnolent, with slight delirium. The temperature rose to 102°, then to $103\frac{5}{10}$° and still later to $106\frac{2}{10}$°; coma ensued and soon afterwards death. All treatment adopted was of no avail; injection of a saline fluid subcutaneously being the only measure which produced the slightest improvement.

He regarded purpura hemorrhagica and scurvy as the same disease, due to the same specific cause, the disease being modified in the latter case by the surroundings and impoverished condition of the patients. Hemorrhages after the ingestion of certain drugs, in the newborn, and with infectious diseases were of neural origin, and probably symptomatic. Purpura simplex and purpura rheumatica were probably identical, the latter being accompanied by joint hemorrhages.

An animated discussion followed the reading of the paper, but little new light was thrown on this obscure subject.

Prof. Delafield remarked that the whole subject of the blood vessels and their contents was still enshrouded with difficulties, and the reason why the vessels sometimes retained their contents, and at other times released them wholly, or in part, was yet unexplained.

Dr. W. P. Northrup related the history of a case of purpura hemorrhagica in a child only eighteen months old. The disease began a month before death, with swelling of the right knee, and a rise of temperature to 101 degrees. Nothing followed for a week, when the gums became swollen and red, and the right eyelid became swollen. One week later other swellings developed, black passages followed and soon afterwards death. At the autopsy, it was found that there had been a hemorrhage between the periosteum and the bone along the shaft of the femur. A similar condition was found on the tibia.

Dr. L. E. Holt added the history of another case in a child only six months old. In this case the first appearance of disease was an occipital bloody tumor, which began as an ecchymotic spot fourteen days before death. Eight days later a few spots appeared on the face, and a hemorrhage took place into the ear. Bloody stools followed in a few days, with bloody vomit and urine. Death was preceded by fever, delirium and coma.

### KOCH'S CURE FOR CONSUMPTION.

The great discovery by Koch of a remedy for tuberculosis is exciting the most intense interest here. The last number of the *Medical News* contains a full translation of Koch's article which has just been published. The exact nature of the remedy is not stated, but it is described as a brownish transparent fluid. It is without effect when introduced into the stomach, and is to be used subcutaneously. The point of injection which has been found most suitable is the skin of the back, between the shoulder blades and the lumbar region. The reaction after the remedy was found to be much more marked in man than in the lower animals. Calculated by body weight one fifteen-thousandth part of the quantity which has no appreciable effect on the guinea pig, acted powerfully on the human being. The symptoms observed by Koch, after an injection into his own arm, were pain in the limbs and fatigue, beginning three or four hours after the injection. These were followed by an inclination to cough and difficulty in breathing, which increased on the fifth day and were unusually severe. A chill followed which lasted nearly an hour, and at the same time there was nausea, vomiting and a rise of temperature. These symptoms all abated in twelve hours, with the exception of a feeling of fatigue and pain in the limbs, which continued for a few days, during which the site of the injection remained slightly painful and red. These effects followed a dose of 0.25 cubic centimeters.

In tuberculosis subjects, however, a severe general reaction followed a much smaller dose. After a dose of 0.01 c. c., there resulted fever preceded by rigors, pain in the limbs, coughing, great fatigue, and often sickness and vomiting. A slight icteroid discoloration was observed in several cases, and occasionally an eruption like measles covered the chest and neck. The attack began four or five hours after the injection, and lasted twelve or fifteen hours. As soon as it was over the patient felt comparatively well.

The local effect of the remedy on tuberculosis lesions was very important and striking. In cases of lupus, in which the effect was visible, the injection under the skin of the back a long distance from seat of disease, was followed by swelling and redness of the lupus patch. This increased during the fever period, sometimes to a high degree, in which case the patch became brownish and necrotic in places. After the fever subsided the swelling gradually diminished. A soft deposit was then to be seen over the seat of disease; this soon changed to a crust which fell off in two or three weeks—sometimes after only one injection—leaving a clean red cicatrix behind.

In cases of tuberculosis of glands, bones, joints, etc., swelling, increased sensibility, and redness of the superficial parts were noticed. In phthisis cases the local reaction was not so apparent, unless the increased cough and expectoration be considered as pointing to a local reaction.

The therapeutic effect in the lupus cases was usually a complete cure, sometimes after one injection, but oftener after several. In cases of phthisis in the early stage every symptom disappeared and the patients might be pronounced cured. Where cavities had developed, great improvement resulted, and only in cases in which large cavities existed did no improvement occur.

The diagnostic value of the remedy is considered by Koch to be very great, the greater susceptibility of tuberculous patients, making possible the discovery of obscure and imperfectly eradicated lesions. WM. L. RUSSELL.

---

PYOXTANINUM CÆRULEUM AND AUREUM IN SOFT CHANCRES AND SYPHILITIC AFFECTIONS.

Our therapeutic armamentarium has lately received two additions which will prove, to all appearances, of a first-rate practical importance; they are *Pyoxtaninum* ("pus-destroyer") *cæruleum* and P. *aureum*, some aniline dyes patented by Professor Stilling and Dr. Merk, both of the substances being unusually powerful and, at the same time, seemingly innocuous antiseptic and "anti-pus" agents (*vide* the *Wiener klinische Wochenschrift*, 1890, May 15). To verify the discoverer's (Prof. Stilling) and patentees' eulogistic statement, Dr. Peterson, of St. Petersburg, has undertaken clinical experiments on forty-eight patients, suffering from soft chancres (20), hard chancres (8), gummous ozæna (4), gummous ulcers of hard palate, pharynx, leg, buttock (5), syphilitic ecthyma, syphilitic keratitis, etc. The remedies were employed in the shape of *a.* pencil made of pure blue or yellow pyoxtanin; *b.* mixtures for powdering prepared of pyoxtaninum and talc (2 to 1,000); *c.* aqueous solutions of pyoxtaninum (1 to 2,000, 1000-100.) The author arrives at the following conclusions: 1. Both blue and yellow pyoxtaninum, in any form, undoubtedly develop powerful antiseptic effects; being by no means inferior to iodoform, they offer the advantage of being quite odorless. 2. They give excellent results both in soft chancres and gummous ulcers, as well as in syphilitic eye diseases. 3. They do not cause any local pain or irritation, and, in fact, appear to be free from any taste or unpleasant accessory effects. 4. The drugs fairly promise to become as widely popular as cocaine. 5. The dyes, however, easily stain the skin and clothes, hence they should not be given the patient himself. The practitioner may easily remove such stains from his clothes by means of dilute hydrochloric or nitric acid.—*British Journal of Derm., November*, 1890.

